# Supplementary material for: MetaQTL: a package of new computational methods for the meta-analysis of QTL mapping experiments
Source: BMC Bioinformatics. 2007 Feb 8;8:49. doi: 10.1186/1471-2105-8-49 (PMC1808479; doi:10.1186/1471-2105-8-49)
Supplement: Additional File 7 — MetaQTL Package : jar file and tutorial. This Zip archive contains both the MetaQTL JAR file and the files of the tutorial. [file 1471-2105-8-49-S7.zip › org.inra.metaqtl/doc/allclasses-frame.html]

All Classes


**All Classes**
  

A2Xml
  
AlleleContainer
  
AnchoredElement
  
BioCrossTypes
  
BioEntity
  
BioEntityAdapter
  
BioEntityBean
  
BioEntityCollection
  
BioEntityCollectionAdapter
  
BioEntityCollectionBean
  
BioEntityContainer
  
BioEntityFactory
  
BioEntityNameComparator
  
BioLocusTypes
  
BioUtilities
  
ChromAxe
  
ChromCluster
  
ChromGraph
  
ChromLayer
  
Chromosome
  
ChromosomeAdapter
  
ChromShape
  
ClustAlgorithm
  
CMarkerSequence
  
CmdLineParser
  
CmdLineParser.IllegalOptionValueException
  
CmdLineParser.NotFlagException
  
CmdLineParser.Option
  
CmdLineParser.Option.BooleanOption
  
CmdLineParser.Option.DoubleOption
  
CmdLineParser.Option.IntegerOption
  
CmdLineParser.Option.LongOption
  
CmdLineParser.Option.StringOption
  
CmdLineParser.OptionException
  
CmdLineParser.UnknownOptionException
  
CmdLineParser.UnknownSuboptionException
  
CMSAlgorithm
  
ConsMap
  
CrossPopulation
  
DubiousLocusFactory
  
EMAlgorithm
  
EMCriteria
  
EMResult
  
EMResultFactory
  
EstimatedLocusPosition
  
GeneticMap
  
GeneticMapBean
  
GeneticMapBeanAdapter
  
GeneticMapProperties
  
GraphFactory
  
GraphShape
  
GroupContainer
  
HClustAlgorithm
  
*IBioAdaptable*
  
*IBioAdapter*
  
*IBioAllele*
  
*IBioConstants*
  
*IBioCross*
  
*IBioCrossTypes*
  
*IBioEntity*
  
*IBioEntityTypes*
  
*IBioGenome*
  
*IBioGenomeProperties*
  
*IBioIndividual*
  
*IBioIndividualTree*
  
*IBioLGroup*
  
*IBioLGroupProperties*
  
*IBioLocus*
  
*IBioLocusProperties*
  
*IBioOntology*
  
*IBioOntologyTerm*
  
*IBioPopulation*
  
*IGeneticMapProperties*
  
*ILGroupPosition*
  
*IMetaAlgorithm*
  
*IMetaQtlConstants*
  
Individual
  
IndividualContainer
  
InfoMap
  
*IOntologyTermProperties*
  
Layer
  
LGroup
  
LGroupBean
  
LGroupBeanAdapter
  
LGroupPosition
  
Locus
  
LocusBean
  
LocusBeanAdapter
  
LocusContainer
  
LocusPositionComparator
  
LocusProperties
  
MapChartFactory
  
MapCluster
  
MapDBFactory
  
MapMakerFactory
  
MappingFunction
  
MapRawFactory
  
MapTabFactory
  
MapView
  
Marker
  
MetaAlgorithm
  
MetaChrom
  
MetaDB
  
MetaDico
  
MetaDicoFactory
  
MetaGraph
  
MetaGraphPar
  
MetaGraphParFactory
  
MetaMain
  
MetaMap
  
MetaMapAdapter
  
MetaMapAlgorithm
  
MetaMapSummaryFactory
  
MetaQtlAnalysis
  
MetaQtlAnalysisAdapter
  
MetaQtlAnalysisFactory
  
MetaQtlAnalysisModelFactory
  
MetaQtlAnalysisSummaryFactory
  
MetaQtlData
  
MetaQtlModel
  
MetaQtlModelFactory
  
MetaQtlResult
  
MMapView
  
MQTLView
  
MultiFitResult
  
NumberFormat
  
NumericalUtilities
  
*NumericalUtilities.Function*
  
*NumericalUtilities.NRFunction*
  
OffspringManager
  
Ontology
  
OntologyBean
  
OntologyBeanAdapter
  
OntologyTabFactory
  
OntologyTerm
  
OntologyTermBean
  
OntologyTermBeanAdapter
  
PaletteColor
  
Population
  
PropertyBean
  
QTL
  
Qtl
  
QtlAdapter
  
QTLClust
  
QtlClustAlgorithm
  
QTLClustInfo
  
QtlLayer
  
QtlLayerBuffer
  
QTLModel
  
QtlPartition
  
QtlPartitionAdapter
  
QtlPartitionLayer
  
QTLProj
  
QtlProjAlgorithm
  
QtlShape
  
QTLTree
  
QtlTreeAlgorithm
  
QtlTreeLayer
  
QtlUnit
  
StaticLocusPosition
  
SVDAlgorithm
  
TabulatedReader
  
TabulatedWriter
  
Tree
  
TreeFactory
  
TreeNode
  
Xml2A
  
XmlBioEntitiesFactory
  
XmlBioEntityFactory
  
XmlGeneticMapFactory
  
XmlOntologyFactory
